# Altered platelet reactivity, coagulation, endothelial and inflammatory markers early after smoking cessation verified with cotinine plasma concentration

**DOI:** 10.1007/s11239-023-02819-5

**Published:** 2023-05-04

**Authors:** Bogumił Ramotowski, Anetta Undas, Andrzej Budaj

**Affiliations:** 1 Department of Cardiology, Centre of Postgraduate Medical Education, Warsaw, Poland; 2 Institute of Cardiology, Jagiellonian University Medical College, Cracow, Poland

**Keywords:** Smoking cessation, Platelet reactivity, Percutaneous coronary intervention, P-selectin, Antiplatelet therapy

## Abstract

**Background/Introduction**: Cigarette smoking is a potent modifiable risk factor for coronary artery disease (CAD). However, little is known about alterations to prothrombotic state and platelet reactivity early after smoking cessation following percutaneous coronary interventions (PCI). **Purpose**: We investigated alterations to platelet reactivity, coagulation and markers of platelet, endothelial, inflammatory and coagulation activation in clopidogrel-treated patients with CAD after PCI before and after smoking cessation. **Methods**: Smoking patients aged 18 years or older at least 30 days after PCI were recruited and encouraged to quit the habit. At baseline and at 30 days, we measured platelet reactivity with VerifyNow system, thrombomodulin, P-selectin, platelet factor 4 (CXCL4/PF4), citrullinated histone H3 (H3cit) and cotinine level. **Results**: Among 117 patients, 84 patients (72%) at a median age of 60.5 years (40 [interquartile range 30–47] pack-years) completed a 30-day follow-up. At day 30, 30 (35.7%) patients stopped smoking with cotinine level < 50 ng/ml. Baseline characteristics were similar in both groups. In smoking quitters a change in platelet reactivity was larger (Δ platelet reactivity units (PRU) 19 [2, 43] vs. -6 [-32, 37], p = 0.018), along with a change in P-selectin concentration (-11.82 [-23.62, 1.34] vs. 7.19 [-14.24, 17.19] ng/ml, p = 0.005). Positive correlations was noticed between cotinine and both P-selectin ( r = 0.23, p = 0.045) and CXCL4 (r = 0.27, p = 0.02). **Conclusion**: After smoking cessation in CAD patients following PCI an increase in platelet reactivity and a decrease in P-selectin levels were observed. The risk of thrombotic complications post PCI might be paradoxically enhanced among patients who stopped smoking.

## Introduction

Cigarette smoking is a potent modifiable risk factor for coronary artery disease (CAD) in patients undergoing percutaneous coronary intervention (PCI) [1]. Cigarette smoking is associated with major changes in the platelet, endothelial, inflammatory, and coagulation responses. Although the precise mechanism underlying smoking-associated impact on CAD remains to be established, generally smoking generates a prothrombotic state involving enhanced platelet activation, increased thrombin generation and unfavorable fibrin clot properties at least in part associated with low-grade inflammation [2]. The active and secondhand smoking effects are well established and the long-term effects of smoking cessation are well known [3], but the changes that occur early after smoking cessation, especially in the setting of PCI with subsequent clopidogrel therapy, remain unclear. The early period after PCI has the highest risk of thrombotic events [4]; therefore, it is crucial to observe changes in platelet and thrombotic factors after smoking cessation. These changes are further complicated by the administration of acetylsalicylic acid (ASA) and the P2Y12 inhibitor clopidogrel to patients with CAD early after PCI. Higher levels of thromboxane metabolites are measured in current smokers and decrease after smoking cessation in patients treated with ASA [5, 6]. Data on the effect of clopidogrel in active and former smokers yielded inconsistent results [7]. Despite the devastating long-term effects of smoking in patients with CAD after PCI, smoking has been shown to enhance the effects of clopidogrel [8]. Clopidogrel is a prodrug metabolized by the liver CYP complex with CYP1A2, which is activated by polycyclic aromatic acids derived from cigarette smoke [9]. Therefore, active smokers treated with clopidogrel achieve higher levels of on-treatment platelet inhibition [10]. This effect may contribute to the clinical smoking paradox [11]. This effect is also reversed after smoking cessation, which may be defined as the smoking cessation paradox [12, 13].

Platelet aggregation, both stimulated and spontaneous, is increased in active smokers [14]. After smoking, platelets are more responsive to lower doses of ADP. An increase in platelet thrombus formation has also been observed as an acute effect of smoking in response to thrombin [15]. Plasma concentrations of platelet activation markers, such as platelet factor 4 (PF4), B-thromboglobulin and platelet activating factor (PAF), are increased compared to that of non-smokers. A small reduction in PF4 concentration has been observed at 12 months after smoking cessation [16]. P-selectin, a component of the α-granule membrane, is expressed and secreted on platelet surfaces following platelet activation [17]. Because P-selectin is shed from the platelet surface, most plasma P-selectin, which can be measured in the blood plasma, is of platelet origin. Compared with non-smokers, active smokers have higher platelet P-selectin expression and a higher plasma level of soluble P-selectin levels [18]. PF4 is the most abundant α-granule protein released by activated platelets, and it is reported to affect various aspects of thrombosis, including potentiated platelet aggregation, and inhibition of thrombin inactivation [19]. Smoke exposure is related to endothelial dysfunction in patients with CAD [20]. Thrombomodulin serves as an endothelial cell surface receptor for thrombin. Soluble thrombomodulin is also an indicator of endothelial cell activation; however, cigarette smoke extracts decrease thrombomodulin receptor expression [21]. Several studies have reported that the level of thrombomodulin in smokers is elevated, but data regarding the changes in thrombomodulin are conflicting, without significant changes in long-term observation [16]. The proinflammatory effects of smoking include increased levels of fibrinogen, C-reactive protein (CRP), and interleukin-6 [22]. Neutrophil extracellular traps (NET) are involved in the immune inflammatory response. NET is a meshwork of DNA fibers composed of histones and antimicrobial proteins and plays a substantial role in thrombus formation, mainly due to the stimulation of platelets by histones [23]. Citrullinated H3 (H3cit), a key component of ET, is a specific biomarker for ETs formation [24]. Extracellular trap formation is linked to prothrombotic state [25] in type 2 diabetes mellitus and the severity of coronary stenosis [26] and the risk of adverse clinical outcomes in stable coronary artery disease [27]. Other markers, such as double-stranded DNA levels, are elevated among smokers versus that in non-smokers with CAD [27]. There have been no published studies as to whether NETosis reflected by increased H3cit could contribute to the prothrombotic state in smokers and changes early after smoking cessation.

To the best of our knowledge, there are no data regarding changes in the factors influencing the coagulation system early after smoking cessation in patients with CAD after PCI. Therefore, we investigated the effect of smoking cessation on platelet reactivity after clopidogrel treatment as well as the markers of platelet, endothelial, proinflammatory, and coagulation activation. We hypothesized that in patients after PCI in early weeks following smoking cessation prothrombotic responses would be decreased but, paradoxically, clopidogrel effect on platelet reactivity may be attenuated.

## Materials and methods

### Patients

We conducted a prospective, observational study of clopidogrel-treated smoking CAD patients who were encouraged to stop smoking. Between 2014 and 2016, consecutive patients with chronic and acute coronary syndromes admitted to the catheterization laboratory in Grochowski Hospital, Warsaw, Poland, were screened for active smoking. Patients aged 18 years or older who smoked at least ten cigarettes per day, at least 30 days after successful PCI with stent placement, and underwent treated with 75 mg clopidogrel and 75 mg acetylsalicylic acid (ASA) once daily, were included in the study and were encouraged to quit the habit. Exclusion criteria were as follows: current anticoagulation, end-stage kidney disease, >3x alanine aminotransferase level, platelet count < 100,000 or > 500,000/mm3, hematocrit level < 30% or > 50%, used strong CYP inhibitors or inducers and the pregnant. Comorbidities were defined as follows: diabetes mellitus as the use of insulin or oral hypoglycemic agents, end stage kidney disease as glomerulal filtration rate (GFR) less than 15 ml/min, arterial hypertension as blood pressure at rest ≥ 140/90 or taking antihypertensive drug, hyperlipidaemia as total cholesterol level > 190 mg/dl or receiving hypolipidemic agents.

Venous blood samples were collected at baseline and 30-day visits using a 21-gauge needle and a 2 mL Vacuette (Greiner Bio-One, Kremsmünster, Austria) coagulation tube with sodium citrate (3.2%) for coagulation, and VerifyNow assay, and a 4 mL Vacuette Serum Clot Activator tube for serum biochemical measurement. Samples were collected 1 h after administration of the morning dose of clopidogrel. Smoking prior to the blood draw was left to the discretion of patients. The first 2–4 mL of blood were discarded to avoid spontaneous platelet activation. Samples were processed for coagulation and platelet function analysis within 2 h after the blood was drawn. Serum levels of cotinine (Calbiotech, California, USA), thrombomodulin (assay range 62.5–4000 pg/mL), P-selectin (0.8–50 ng/mL), PF4 (0.8–50 ng/mL) (all from R&D, Minneapolis, USA), and citH3 (Cayman Chemical, Ann Arbor, Michigan, USA) (0.15-10 ng/ml), were assayed in duplicate using enzyme-linked immunoabsorbent assays according to the manufacturer’s instructions. The interassay coefficient of variation was < 7%. Smoking cessation was defined as a cotinine concentration of < 50 ng/mL [28, 29].

VeifyNow (Accriva Diagnostics, San Diego, California, United States) P2Y12 is a turbidimetric-based assay. The probes were processed for at least 15 min after collection. The results were reported as P2Y12 reaction units (PRU), with a level > 208 PRU indicating high on-treatment platelet reactivity reference range for dual antiplatelet therapy [4, 30].

### Ethical considerations

The study complied with the principles of the Declaration of Helsinki. Approval numbers 16/PB/2014 and 10/PB-A/2015 were provided by the Ethics Committee of the Centre of Postgraduate Medical Education. Informed consent was obtained from all patients. The study was retrospectively registered at ClinicalTrials.gov with a NCT040784702 trial identifier as part of a larger project.

### Interventions

All the patients were advised to stop smoking. All patients received low-intensity counseling that focused on the benefits of quitting, particularly on the reduced risk of cardiovascular disease.

### Statistical methods

Baseline characteristics are presented as the mean and standard deviation (SD) or median and interquartile range (IQR) for normally or non-normally distributed continuous data, respectively, and as the frequency for categorical data. The Shapiro-Wilk test was used to assess data normality. A multivariable logistic regression model was applied to investigate covariates associated with changes of PRU and P-selectin level and PRU > 208 level at follow-up visit. The following variables were included in the model: sex, age, recent ACS, pack-years. All analyses were performed using Stata 14.1 software (StataCorp, LP, College Station, Texas, USA).

## Results

Among 117 patients who attended the baseline visit, 84 completed a 30-day follow-up. The median [IQR] age of the patients was 60 [55–65] years; the median body mass index was 27.55 [25.2–30.9] kg/m^2^, and the median load of smoking was 40 [30–47] pack-years. At the follow-up visit, 30 (35.7%) patients had stopped smoking, as confirmed by low cotinine concentrations (study group), and 54 patients had continued smoking (control group). The baseline characteristics, including the proportion of ACS patients, were similar in both groups (Table 1).

**Table 1:** Baseline characteristics of the study and control groups

Variable	Study group (N = 30)	Control group (N = 54)	P
Age (years) median [IQR]	61 [55–66]	62.5 [58–65]	0.64
Men, n (%)	20 (67)	41 (76)	0.44
Recent ACS, n (%)	12 (40)	26 (48.15)	0.47
Prior MI, n (%)	8 (26.7)	15 (27.8)	0.91
Prior PCI, n (%)	4 (13.4)	4 (7.4)	0.37
Diabetes, n (%)	9 (30)	11 (20.4)	0.32
Hypertension, n (%)	27 (90)	42 (77.8)	0.16
Hyperlipidemia, n (%)	32 (76.2)	45 (77.8)	0.9
Secondhand smoking, n (%)	22 (73.3)	39 (72.2)	0.9
Cigarette smoking (pack-years, median [IQR]	40 [30–47]	35 [25–48]	0.7
Statin treatment, n (%)	29 (96.7)	53 (98.1)	0.36
Proton pump inhibitor treatment, n (%)	12 (40)	30 (55,5)	0.15
Platelet count (n/µl), median [IQR]	223.2 [189.3-274.8]	244.15[209.9-279.3]	0.38
HGB (g/dl), median [IQR] Creatinine level (g/dl), median [IQR]	14.5 [13.6–15.4] 0.94 [0.84-1]	14.55 [13.35–14.9] 0.84 [0.75-1]	0.5 0.05

ACS, acute coronary syndrome; MI, myocardial infarction; PCI, percutaneous coronary intervention; Ns, not significant. All data were given as median [IQR].

In the study group as compared to the control group, at day 30 a change in platelet reactivity was higher (Δ platelet reactivity units (PRU) 19 [2, 43] vs. -6 [-32, 37], p = 0.018), along with a change in P-selectin concentration (-11.82 [-23.62, 1.34] vs. 7.19 [-14.24, 17.19] ng/ml), p = 0.005). The change in CXCL4/PF4 was numerically higher in the smoking cessation group compared with that in the control group (-501.4 [-2440.2, 846.7] ng/mL vs. 154.9 [-1220, 2289.7] ng/mL, p = 0.089). The changes in the concentrations of thrombomodulin and citH3 did not differ significantly between the groups, nor did the levels of CRP, fibrinogen, APTT and prothrombin index. (Table 2).

**Table 2:** Changes in platelet, endothelial, inflammatory and coagulation markers at follow-up visit

Variable	Study group (N = 30)	Control group (N = 54)	P
Platelet reactivity units (PRU), median [IQR]	19 [2, 43]	-6 [-32, 37]	P = 0.018
P-selectin ng/ml, median [IQR]	-11.82 [-23.62, 1.34]	7.19 [-14.24, 17.19]	P = 0.005
CXCL4/PF4 ng/ml, median [IQR]	-501.4 [-2440.2, 846.7]	154.9 [-1220, 2289.7]	P = 0.089
Thrombomodulin pg/ml, median [IQR]	22.85 [-184.5, 262.4]	31.2 [-195.8, 219.4]	P = 0.708
CitH3 ng/ml, median [IQR]	-0.26 [-1, 0.07]	-0.08 [-0.74, 0.21]	P = 0.275
CRP mg/l, median [IQR)	0.1 [-0.25,0.6]	-0.2 [-1.2,0.5]	P = 0.262
Fibrinogen mg/ml, median [IQR]	-11.5 [-45, 36]	-18 [-52,46]	P = 0.957
APTT s, median [IQR]	-0.25 [-1.55, 1.10]	-0.45 [-1.20, 1.3]	P = 0.742
Prothrombin index %, median [IQR]	1.5 [-2.5, 7]	-2 [-6, 3]	P = 0.076
Platelet count (10^3^/µl), median [IQR]	223.2 [189.3-274.8]	244.15 [209.9-279.3]	P = 0.38

IQR, interquartile range; PRU, platelet reactivity units; CXCL4/PF4, platelet factor 4; cit H3, citrullinated histone H3, APTT, activated partial thromboplastin time; CRP, C-reactive protein. All data were given as median [IQR].

Cotinine levels were not associated with any of the factors at the baseline visit, but there were positive correlations between cotinine and both P-selectin ( r = 0.23, p = 0.045) and CXCL4 (r = 0.27, p = 0.02) at the follow-up visit. We did not find any correlation between PRU change and P-selectin change at follow-up visit. The groups of patients after PCI in ACS and CCS did not differ significantly with regard to PRU and P-selectin in univariable and multivariable analysis.

## Discussion

Our study confirms platelet reactivity changes following clopidogrel treatment, with a parallel observation of changes in platelet, endothelial, proinflammatory, and coagulation activation markers. Our data show that despite the decreased effect of clopidogrel on P2Y12 receptor, smoking cessation diminished platelet activation measured by P-selectin early after smoking cessation, without major changes in the concentration of other factors.

Early after stent implantation, it is crucial to achieve adequate platelet inhibition with clopidogrel in patients with acute and chronic coronary syndrome. Large trials in this population revealed that the short-term prognosis of smokers treated with clopidogrel was better than that of non-smokers [31]. Nevertheless, most of the trials did not follow changes in the smoking status of clopidogrel-treated patients. High platelet reactivity (HPR) measured using VerifyNow, is related to an increased risk of thrombotic complications after PCI [4]. In our trial, we found that smoking cessation was related to almost a 19 PRU increase versus a 6 PRU decrease in the smoking continuation group. According to previous studies every 10-unit increase in PRUs may raise the primary endpoint by 4% (hazard ratio [HR]:1.04, 95% confidence interval [CI]:1.03–1.06, p < 0.0001) [32].

The normalization of various factors induced by smoking lasts from months for endothelial coagulation [16] to years for inflammatory factors [33]. We found only modest normalization of prothrombotic factors in our trial within a short period after smoking cessation. Soluble P-selectin levels decreased by almost 12 ng/mL versus a decrease of 7 ng/mL in patients who continued smoking. P-selectin seems to be one of the most important markers of platelet activation [34] and might directly promote thrombosis. It is also a predictor of adverse cardiovascular events [34]. The decrease in P-selectin after smoking cessation has been previously reported [35] but our study adds data regarding patients on P2Y12 with an objective measure of smoking cessation. The data regarding clopidogrel treatment and level of soluble P-selectin are conflicting. The relation of response to clopidogrel treatment was not correlated with soluble P-selectin level so far [36], but in another study patients with clopidogrel resistance had higher levels of P-selectin [37] .

PF4 is a specific alpha granule protein released by activated platelets. In our study, the change in PF4 was greater after smoking cessation. PF4 concentration was also correlated with cotinine levels at the follow-up visit, but not at the baseline visit. This is consistent with the findings of Capponetto et al., who found that the median concentration of PF4 decreased at 2, 6, and 12 months after smoking cessation, with significant changes at 6 and 12 months. In aforementioned study the number of pack-years was positively correlated with PF4 concentrations [16]. Our data suggest that the decrease in PF4 was faster.

We did not find a significant difference in thrombomodulin concentrations at the follow-up visit. This suggests that the effect of smoking cessation on thrombomodulin might be delayed.

This is the first study to assess NETosis marked by H3cit in active smokers and after smoking cessation. We did not find any correlation between citH3 and cotinine levels at baseline and follow-up visits. There was also no significant difference in citH3 concentration after smoking cessation. Other markers of inflammation, such as CRP did not differ between the groups.

We also did not revealed any substantial changes in fibrinogen. Fibrinogen level is increased in active smokers [2] but it takes years to normalize after smoking cessation [3] therefore change in early period after smoking cessation might not be visible.

In most previous trials, smoking was assessed only based on self-report, hence the rates of smoking cessation might be underestimated [38]. Plasma cotinine level is an objective and reliable marker of smoking and smoking cessation, which may vary depending on the population or secondhand smoking status [29, 39].

Our findings in patients on clopidogrel therapy suggest that early after smoking cessation, only platelet activation markers decrease, without major changes in endothelial, inflammation and coagulation markers. This trend was accompanied with the decrease in P2Y12 platelet inhibition measured using VerifyNow. The total effect of the changes after smoking cessation remains a matter of investigation.

Our study has several limitations. First, because of the nature of addiction and ethics, we could not arrange a randomized study; therefore, it is observational in nature. Our cut-off level for cotinine concentration was relatively high, but it was based on previous experience with a population with a high load of smoking and a substantial number of second-hand smokers. Furthermore, we analyzed only the selected markers of the prothrombotic state and measured platelet reactivity to clopidogrel using only one method, P2Y12 VerifyNow, however, the method was validated and widely used in clinical trials.

## Conclusion

In the early period after smoking cessation, patients with CAD who underwent PCI and were treated with clopidogrel and ASA, had an increase in platelet reactivity, a decrease in P-selectin, and a trend towards lower PF4 levels. No significant changes were observed in the other markers. It is speculated that the risk of thrombotic complications might be paradoxically enhanced among such patients after smoking cessation despite dual antiplatelet therapy.

## Abbreviations

PCI: Percutaneous coronary intervention
CAD: Coronary artery disease
ASA: Acetylsalicylic acid
PAF: Platelet activating factor
CXCL4/PF4: Platelet factor 4
NET: Neutrophil extracellular traps
H3cit: Citrullinated H3
APTT: Activated partial thromboplastin time
CRP: C-reactive protein
SD: Standard deviation
IQR: Interquartile range
